# Diet-Induced Obesity Disrupts Trace Element Homeostasis and Gene Expression in the Olfactory Bulb

**DOI:** 10.3390/nu12123909

**Published:** 2020-12-21

**Authors:** Melissa S. Totten, Derek M. Pierce, Keith M. Erikson

**Affiliations:** Department of Nutrition, School of Health and Human Sciences, University of North Carolina at Greensboro, Greensboro, NC 27412, USA; dmpierc2@uncg.edu (D.M.P.); kmerikso@uncg.edu (K.M.E.)

**Keywords:** obesity, diet, high fat, olfactory bulb, sex, strain, alpha-synuclein, neurodegenerative disease, iron, manganese

## Abstract

The aim of this study was to determine the impact of diet-induced obesity (DIO) on trace element homeostasis and gene expression in the olfactory bulb and to identify potential interaction effects between diet, sex, and strain. Our study is based on evidence that obesity and olfactory bulb impairments are linked to neurodegenerative processes. Briefly, C57BL/6J (B6J) and DBA/2J (D2J) male and female mice were fed either a low-fat diet or a high-fat diet for 16 weeks. Brain tissue was then evaluated for iron, manganese, copper, and zinc concentrations and mRNA gene expression. There was a statistically significant diet-by-sex interaction for iron and a three-way interaction between diet, sex, and strain for zinc in the olfactory bulb. Obese male B6J mice had a striking 75% increase in iron and a 50% increase in manganese compared with the control. There was an increase in zinc due to DIO in B6J males and D2J females, but a decrease in zinc in B6J females and D2J males. Obese male D2J mice had significantly upregulated mRNA gene expression for divalent metal transporter 1, alpha-synuclein, amyloid precursor protein, dopamine receptor D2, and tyrosine hydroxylase. B6J females with DIO had significantly upregulated brain-derived neurotrophic factor expression. Our results demonstrate that DIO has the potential to disrupt trace element homeostasis and mRNA gene expression in the olfactory bulb, with effects that depend on sex and genetics. We found that DIO led to alterations in iron and manganese predominantly in male B6J mice, and gene expression dysregulation mainly in male D2J mice. These results have important implications for health outcomes related to obesity with possible connections to neurodegenerative disease.

## 1. Introduction

The prevalence of obesity in the United States is steadily increasing and was estimated to affect 42% of the adult population between 2017 and 2018 [1]. Among the numerous adverse health effects associated with obesity, such as increased cholesterol, hypertension, and insulin resistance, obesity is a potential risk factor for incurable neurodegenerative diseases such as Alzheimer’s disease (AD) [2] and Parkinson’s disease (PD) [3]. The risk of developing a neurodegenerative disease depends on a combination of genetics and the environment [4]. For example, genetic mutations in amyloid precursor protein (APP) [5] and alpha-synuclein (aSyn) [3] have been associated with the progressive development of AD and PD, respectively. Environmental exposure to metals such as manganese (Mn) has been linked to the onset of PD [6]. Furthermore, it has been found that specific genetic mutations may predispose individuals to metal toxin sensitivity, increasing their risk for PD [7]. While our laboratory has primarily focused on mechanisms of neurodegenerative processes resulting from environmental exposures to metals (e.g., drinking water) [8,9], given recent data on altered brain metal metabolism due to diet-induced obesity (DIO) [10,11], we sought to determine the mechanisms by which DIO-associated changes in metals can potentially lead to neurodegenerative disease.

Recently in our lab, it was discovered that DIO alters iron (Fe) homeostasis and gene expression in the brain of C57BL/6J (B6J) male mice [10,11,12]. In one study, B6J male mice fed a high-fat diet (HFD) had elevated Fe and an upregulation of aSyn in the midbrain [10]. These preliminary findings suggest that obesity and PD could share signs of neuropathology. PD is characterized by intracellular aggregates of aSyn in the form of Lewy bodies localized in the substantia nigra [13]. Fe is also known to be elevated in the substantia nigra of PD patients, while copper (Cu) is diminished [14]. Interestingly, it has been discovered that aSyn accumulates in the olfactory bulb long before it does in the substantia nigra and is associated with the loss of the olfactory sense [15]. Loss of olfaction is an early symptom of AD [16] and PD [17], and usually occurs years before disease onset. Analysis of postmortem brain tissue revealed that aSyn was accumulated in the olfactory bulbs of male and female PD patients and confirmed Lewy body formation [18,19]. Additionally, the olfactory bulb was determined in autopsy studies to be the first brain region affected by Lewy-type alpha-synucleinopathy [15]. Compared with other brain regions, the olfactory bulb has less protection by the blood–brain barrier and more exposure to the environment through the nasal cavity, providing a potential route for toxins or a beneficial entry for therapeutics [20]. Trace elements have also been found to be altered in the olfactory bulb in AD and PD. For example, in a study using brain tissue from AD patients, Fe and zinc (Zn) were significantly elevated in the olfactory bulb [21]. Furthermore, in a study using postmortem human brain tissues from male and female individuals, Fe concentration was 25% higher in PD olfactory bulbs compared with that in control samples [22]. In this same study, Zn concentration in the PD olfactory bulbs was reduced, although not significantly, and Cu, Mn, and other trace element levels in the olfactory bulbs were unaffected. While trace element homeostasis in the context of neurodegenerative disease has been studied extensively in the substantia nigra using human and animal models [14,23], it has not received the same attention in the olfactory bulb. Thus, our study investigates the impact of DIO on trace element homeostasis in the olfactory bulb, including the expression of select genes related to trace element neurobiology, neurodegeneration, and obesity. 

Sex and genetics are important biological factors to consider in the study of DIO. The inclusion of both males and females in research studies is a key initiative proposed by the National Institutes of Health [24]. Supporting evidence for sex differences in response to a HFD and a state of DIO can be found in several recent human and rodent obesity reports [25,26,27]. For example, in a study using male and female rats, males fed an HFD were found to be more prone to brain oxidative stress and trace element disruptions than females [26]. Another study that examined neurodegeneration and brain connectivity in a group of patients with probable AD found that the effect of obesity on brain metabolism was more significant in females versus males [27]. Furthermore, genetic background is an important biological variable, as several recent studies have indicated that strain or genetic variation can influence the effect of DIO on disease risk [28] and gene expression modification [29]. These human and animal studies highlight the significant influence of sex and genetics on biological alterations and eventual health outcomes that can be triggered by DIO. Our study included male and female B6J and DBA/2J (D2J) mouse strains to address sex and genetic factors. These strains display differential traits when exposed to various stresses and are frequently used in the study of neuroscience [30]. Furthermore, the B6J and D2J strains have previously been validated as effective DIO models [31,32,33].

The objective of this study was to determine the impact of DIO on trace element dysregulation and gene expression alterations in the olfactory bulb and to identify potential interaction effects between diet, sex, and strain. Trace elements Fe, Mn, Cu, and Zn were selected for this study based on their frequent association with neurodegenerative disease [34]. We hypothesized that DIO would cause alterations in Fe, Zn, and aSyn gene expression in the olfactory bulb, as these physiological changes have been implicated in the pathogenesis of PD and AD [15,21,22] and may share similar pathogenic mechanisms or neurobiological disruptions to those created by a state of DIO.

## 2. Materials and Methods 

### 2.1. Animals and Diet

Male and female C57BL/6J (B6J) mice (*n* = 36) and DBA/2J (D2J) mice (*n* = 36) were purchased from the Jackson Laboratory (Bar Harbor, ME, USA) at postnatal day 21. After 3 days of acclimation in the animal care facility, the mice were randomly assigned a control low-fat diet (LFD) with 10% kcal fat/g (Research Diets, D12450J) or a mineral-matched high-fat diet (HFD) with 60% kcal fat/g (Research Diets, D12492) for 16 weeks as described previously [12]. Both diets met the minimum nutrient requirements for rodents [35]. Each control and treatment group comprised *n* = 9 (Table 1). The number of animals required for each experiment in this study was based on a power analysis using an estimated variance from preliminary studies from our laboratory. The assigned diets were provided as ad libitum feeding with free access to deionized water 24 h/day. During the 16-week diet treatment, the mice were weighed once per week, and food weight was recorded 3 days/week. The mice were housed three per cage with the males and females positioned on opposite sides of a temperature-controlled room (25 °C) maintained on a 12 h light–dark cycle.

This study was conducted in an American Association for Laboratory Animal Care-accredited facility following a protocol approved by the Institution of Animal Care and Use Committee at the University of North Carolina Greensboro. Procedures were performed according to the principles and guidelines established by the National Institutes of Health for the ethical care and use of laboratory animals. 

### 2.2. Tissue Collection

After the 16-week dietary treatment, 44 mice were humanely anesthetized with isoflurane followed by rapid decapitation. The remaining mice were used in a separate study (data reported elsewhere). Brains were dissected sagittally into right and left hemispheres on an ice-cold stainless steel platform. Olfactory bulb tissue was snap-frozen in liquid nitrogen, placed on dry ice, and then stored in a −80 °C freezer until further processing. Right and left hemispheres were randomly assigned for analysis of trace elements or mRNA gene expression.

### 2.3. Trace Element Analysis

Trace elements Fe, Cu, Mn, and Zn were evaluated using graphite furnace atomic absorption spectrometry (Model AA240, Agilent Technologies, Inc., Santa Clara, CA, USA) with concentrations reported as micrograms of metal per gram of protein. Based on a power analysis from our previous studies [10,11], a sample size of *n* = 5 per group was used. Total protein was analyzed using a Pierce™ bicinchoninic acid protein quantitation assay (Thermo Fisher Scientific, Inc., Waltham, MA, USA). Olfactory bulb samples were sonicated in cold radioimmunoprecipitation assay buffer containing protease inhibitors. Homogenates were then digested in ultrapure nitric acid as a 1:1 ratio for 24 h in a sand bath between 60 and 80 °C. Aliquots were diluted with 2% nitric acid before use on the graphite furnace atomic absorption spectrometer. Bovine liver National Bureau of Standards standard reference material, USDC, Washington, DC, USA) digested in ultrapure nitric acid was used as the internal standard. 

### 2.4. RNA Isolation and cDNA Synthesis

RNA was isolated from frozen tissue (*n* = 3–5 per group) with the RNeasy® Plus Mini Kit (Qiagen Inc., Germantown, MD, USA) following the manufacturer’s protocol. RNA concentration and purity were confirmed with a NanoDrop™ 1000 spectrophotometer (Thermo Fisher Scientific, Inc., Waltham, MA, USA). Reverse transcription of RNA was conducted on Applied Biosystems GeneAmp® PCR System 9700 using the Applied Biosystems High-Capacity cDNA Reverse Transcription Kit (Life Technologies, Carlsbad, CA, USA) to prepare 20 μL samples for the thermocycler. The reaction conditions were as follows: 25 °C for 10 min, 37 °C for 120 min, 85 °C for 5 s, and 4 °C holding temperature at completion. The prepared samples were stored at −20 °C until further evaluation. 

### 2.5. Real-Time Polymerase Chain Reaction (RT-PCR)

Relative gene expression was determined by RT-PCR on a 7500 Fast Real-Time PCR System from Applied Biosystems under the following conditions: incubation for 2 min at 50 °C, polymerase activation for 2 min at 95 °C, and 40 cycles of PCR (denature for 3 s at 95 °C and anneal/extend for 30 s at 95 °C). TaqMan™ gene assays were supplied from Life Technologies (Carlsbad, CA, USA) and included the following: *SLC11A2* for divalent metal transporter 1 (DMT1), *SNCA* for alpha-synuclein (aSyn), *APP* for amyloid beta precursor protein (APP), *DRD2* for dopamine receptor D2 (DRD2), *TH* for tyrosine hydroxylase (TH), and *BDNF* for brain-derived neurotrophic factor (BDNF). Each assay was prepared for RT-PCR using Applied Biosystems™ TaqMan™ Fast Advanced Master Mix. The expression of each gene was normalized using 18S as the endogenous control. Normalized cycle threshold (Ct) values were used to determine the interactions and main effects of diet, sex, and strain. The comparative Ct method was used to determine fold change in gene expression comparing LFD (control) with HFD for each sex and strain, or to compare males with females for sex differences.

### 2.6. Statistical Analysis

The effects of diet, sex, and strain on trace element concentration and gene expression were evaluated using a three-way analysis of variance (ANOVA). Statistically significant interaction effects were assessed further for simple main effects. In cases of no interactions, statistically significant main effects were reported. Differences between treatment groups at each level were determined by pairwise comparisons with the application of Bonferroni adjustment. Normality and homogeneity of variance of data were confirmed using the Shapiro–Wilk test and Levene’s test, respectively. If equal variances could not be achieved to perform a three-factor ANOVA, independent *t*-tests were used to compare differences between groups. Statistical significance was accepted at *p* < 0.05, and differences were considered approaching significance between *p* = 0.05 and 0.10. Data are reported as means ± standard error of the mean (SEM). Statistical analysis was performed using IBM SPSS Statistics 26.

## 3. Results

### 3.1. Weight Gain

**Figure 1:**
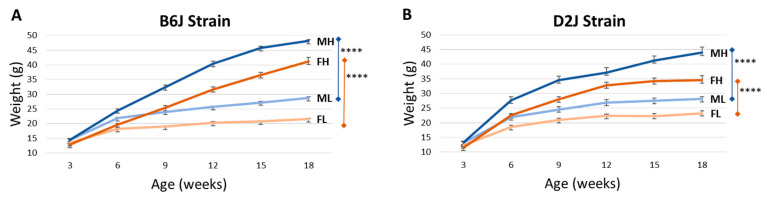
Weight gain by strain. Weight gain throughout the 16-week diet treatment for C57BL/6J (B6J) male and female mice (**A**) and DBA/2J (D2J) male and female mice (**B**). Letter codes are as follows: M = male, F = female, L = low-fat diet, H = high-fat diet. Data are represented as mean ± SEM. **** *p* < 0.0001.

Changes in body weight over the 16-week diet treatment for each strain and sex are shown in Figure 1. The percent weight gain over the 16-week diet treatment was significantly higher for mice in the HFD treatment groups compared with that in the LFD treatment groups for each strain and sex. Since equal variances could not be achieved to run a three-factor ANOVA, comparisons between week 1 weight and week 16 weight were made using independent t tests. B6J males fed an LFD gained 102 ± 17% grams in weight, while those in the HFD group gained 246 ± 26% (t_15_ = 14.019, *p* < 0.0001). For B6J females, mice fed an LFD gained 62 ± 12% weight, and mice fed an HFD gained 225 ± 32% weight (t_14_ = 14.211, *p* < 0.0001). D2J males fed an LFD gained 117% ± 27% weight, while those in the HFD group gained 235 ± 27% weight (t_15_ = 8.951, *p* < 0.0001). The D2J female group fed an LFD gained 88 ± 17% weight, and the HFD group gained 181 ± 37% weight (t_14_ = 6.693, *p* < 0.0001). There was no significant difference in grams of food eaten when comparing the LFD and HFD groups (LFD group = 2.536 g/day ± 0.0717 g/day, HFD group = 2.455 g/day ± 0.0493; t_20_ = 0.940, *p* = 0.358), indicating that any change in trace element concentration would not be due to differences in consumption of dietary minerals. 

### 3.2. Impact of DIO on Trace Elements in the Olfactory Bulb

**Figure 2:**
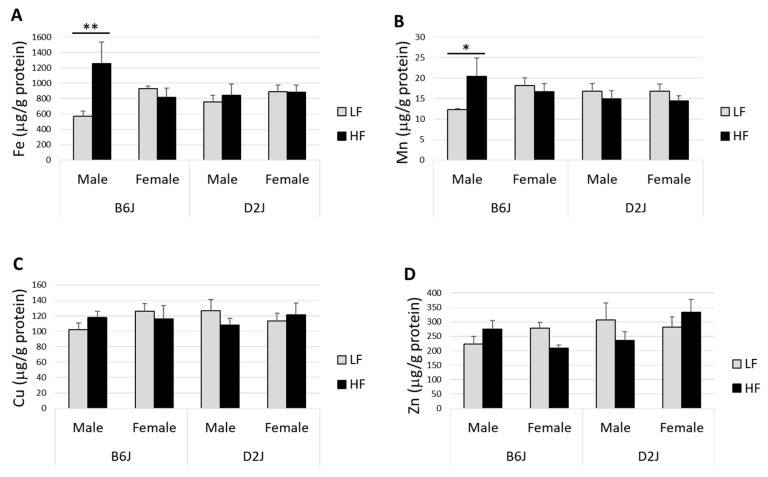
Impact of diet-induced obesity (DIO) on olfactory bulb trace elements. The effects of diet, sex, and strain on olfactory bulb iron (Fe) (**A**), manganese (Mn) (**B**), copper (Cu) (**C**), and zinc (Zn) (**D**) in male and female C57BL/6J (B6J) and DBA/2J (D2J) mice. Male B6J mice fed a high-fat diet have significantly increased levels of both Fe and Mn. Data are represented as mean ± SEM. * *p* < 0.05, ** *p* < 0.01. LF = low fat, HF = high fat.

The impact of DIO on trace elements Fe, Mn, Cu, and Zn in the olfactory bulb is shown in Figure 2. There was a significant two-way interaction between diet and sex on Fe in the olfactory bulb (F_1,29_ = 6.241, *p* = 0.018), with a simple main effect of diet in male mice only (F_1,29_ = 9.169, *p* = 0.005). Male mice (B6J and D2J combined) fed an HFD diet had an overall increase in Fe by 41%, while female mice showed a decrease in Fe by 6%. The impact of diet on Fe in this brain region was greatest for B6J male mice, with an estimated 75% increase in Fe (F_1,29_ = 12.987, *p* = 0.001) (Figure 2A). There were no interaction effects for Mn in the olfactory bulb; however, there was a statistically significant increase in Mn by 50% in B6J male mice fed an HFD (F_1,30_ = 4.675, *p* = 0.039) (Figure 2B). Cu in the olfactory bulb was not significantly impacted by DIO (Figure 2C). The difference in Cu/Zn ratio in B6J females was approaching statistical significance (F_1,31_ = 2.937, *p* = 0.097), with the HFD group showing a 24% higher ratio compared with the LFD group.

**Figure 3:**
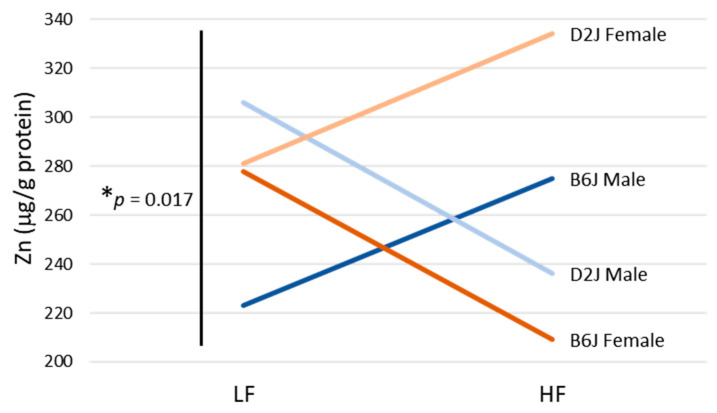
Olfactory bulb zinc (Zn) three-way interaction. Three-way interaction between diet, sex, and strain on Zn in the olfactory bulbs of male and female C57BL/6J (B6J) and DBA/2J (D2J) mice due to diet-induced obesity.

An evaluation of Zn in the olfactory bulb revealed a significant three-way interaction between diet, sex, and strain on Zn concentration (F_1,30_ = 6.329, *p* = 0.017) (Figure 3). Zinc increased by 21% in B6J male mice fed an HFD, but decreased by 28% in B6J female mice. The opposite trend occurred in D2J mice, with a 26% decrease in Zn for D2J male mice fed an HFD, but a 17% increase for D2J females. While this three-way interaction was statistically significant, individual differences based on pairwise diet comparisons were not significantly different due to high variance. 

### 3.3. Impact of DIO on Gene Expression in the Olfactory Bulb

**Figure 4:**
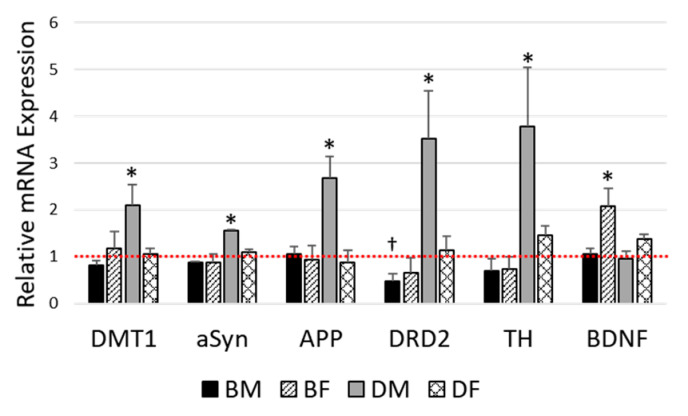
**Diet-induced obesity** impact on olfactory bulb gene expression. Fold change comparisons by diet for these genes are shown for the olfactory bulb. The low-fat diet (LFD) mRNA expression was established as the control (LFD reference = 1 as indicated by the horizontal dashed bar). Fold change compared with each control was determined using the comparative Ct method. Data are represented as mean ± SEM. * *p* < 0.05, † approaching significance (*p* = 0.05–0.10). DMT1 = divalent metal transporter 1, aSyn = alpha-synuclein, APP = amyloid precursor protein, DRD2 = dopamine transporter D2, TH = tyrosine hydroxylase, BDNF = brain-derived neurotrophic factor, BM = C57BL/6J males, BF = C57BL/6J females, DM = DBA/2J males, DF = DBA/2J females.

The expression of mRNA in the olfactory bulb was evaluated for DMT1, aSyn, APP, DRD2, TH, and BDNF. In D2J male mice fed an HFD, there was a statistically significant upregulation in mRNA gene expression for DMT1 by 2.1-fold (F_1,23_ = 4.608, *p* = 0.043), aSyn by 1.6-fold (F_1,22_ = 4.805, *p* = 0.039), APP by 2.7-fold (F_1,23_ = 7.436, *p* = 0.012), DRD2 by 3.5-fold (F_1,19_ = 5.879, *p* = 0.025), and TH by 3.8-fold (F_1,16_ = 6.444, *p* = 0.022) compared with the control LFD (Figure 4). A downregulation of DRD2 in B6J male mice fed an HFD relative to control (approximately 2-fold decrease) was approaching significance (F_1,24_ = 3.868, *p* = 0.064). Furthermore, there were significant diet-by-strain interactions for aSyn (F_1,22_ = 4.726, *p* = 0.041), DRD2 (F_1,19_ = 9.313, *p* = 0.007), and TH (F_1,16_ = 7.213, *p* = 0.016) that revealed a slight downregulation of gene expression in the B6J strain but an upregulation of gene expression in the D2J strain due to DIO.

For the expression of BDNF, there was a diet-by-sex interaction (F_1,24_ = 4.378, *p* = 0.047). Female mice fed an HFD had 1.9-fold higher BDNF expression compared with female mice fed an LFD (F_1,30_ = 7.811, *p* = 0.10), but there was no significant difference in expression for males. Pairwise comparisons revealed that B6J females fed an HFD were impacted the greatest, with an upregulation of BDNF by 2.1-fold (Figure 4). 

Additionally, there were significant sex-by-strain interaction effects for the mRNA expression of DMT1, aSyn, and APP. For DMT1, the effect of sex was significant for B6J mice only (F_1,23_ = 23.708, *p* < 0.0001), with B6J females expressing 3.0-fold higher mRNA compared with B6J males. aSyn showed a similar pattern of a sex effect that was evident in B6J mice only (F_1,22_ = 32.455, *p* = 0.0001), with B6J females expressing 2.4-fold higher mRNA compared with B6J males. The sex-by-strain interaction was different for APP mRNA expression, with a significant sex effect for D2J mice only (F_1,23_ = 158.745, *p* < 0.0001). In this case, D2J males had a 23-fold higher expression level compared with D2J females. 

## 4. Discussion

The goal of this study was to determine the impact of DIO on trace element homeostasis and gene expression in the olfactory bulb, and to identify potential interaction effects between diet, sex, and strain. Using male and female B6J and D2J mice fed either a control LFD or a lard-based HFD for 16 weeks, we discovered that there was a statistically significant diet-by-sex interaction for Fe and a three-way interaction between diet, sex, and strain for Zn in the olfactory bulb. Male mice fed an HFD had increased Fe and Mn, with a striking 75% increase in Fe for B6J males. There was an increase in Zn due to DIO in B6J males and D2J females, but a decrease in Zn for B6J females and D2J males. Male D2J mice fed an HFD showed a significant upregulation of mRNA gene expression for DMT1, aSyn, APP, DRD2, and TH. B6J females fed an HFD had significantly upregulated BDNF expression. Overall, these findings reveal that DIO has a substantial impact on olfactory bulb Fe, Mn, and Zn homeostasis and gene expression in a sex- and strain-dependent manner.

All mice fed a lard-based HFD with 60% kcal fat gained a significant amount of weight compared with mice fed an LFD (Figure 1). This weight gain is consistent with previous reports using B6J and D2J strains for DIO investigations [31,32,33] and previous DIO studies from our lab using male B6J mice [10,11]. The diet treatment was initiated at a young age of approximately 3 weeks old and continued for 16 weeks. Based on the significant weight gain that we observed and measured in both male and female B6J and D2J mice, we can conclude that this diet regimen provided a successful model to study the impact of DIO on trace element homeostasis and gene expression in the olfactory bulb.

DIO had a significant impact on olfactory bulb Fe, Mn, and Zn homeostasis. We discovered a diet-by-sex interaction for Fe, which showed an increase in Fe in males but a decrease in Fe in females due to DIO. The impact of DIO was greatest in B6J males, which showed a 75% higher concentration of Fe (Figure 2A) and a 50% elevation in Mn (Figure 2B) in the HFD group versus the LFD group. Although there were no differences in Cu concentrations in the olfactory bulb due to DIO, the Cu/Zn ratio for female B6J mice was 24% higher for the HFD group compared with that for the LFD group and was trending toward statistical significance (*p* = 0.097). Similar reports of elevated Cu/Zn ratios in blood have occurred previously in cases of autism spectrum disorders [36], aging [37], and inflammation [38]. For Zn, there was a significant three-way interaction between diet, sex, and strain in the olfactory bulb (Figure 3). Concentrations of Zn were increased in B6J males and D2J females but were decreased in B6J females and D2J males by a similar magnitude. While we do not know the specific mechanism(s) as to why this three-way interaction exists for Zn only, we speculate that it could be due to the response of specific genes to DIO. There are some similarities between our trace element results and those of other studies performed in humans with neurodegenerative disease. For example, in a study using postmortem brain tissues, Fe concentration was 25% higher in PD olfactory bulbs compared with controls [22]. In another study using brain tissues from AD patients, Fe and Zn were significantly elevated in the olfactory bulbs [21]. Furthermore, an examination of young adults exposed to high levels of pollution found increased Mn in the olfactory bulbs accompanied by upregulated IL1b mRNA, suggesting that elevated Mn is associated with neuroinflammation in this brain region [39]. Our study provides preliminary evidence that DIO can disrupt trace element homeostasis in the olfactory bulb and could potentially be a contributing factor to the development of neurodegenerative disease.

The influence of DIO on olfactory bulb gene expression was evaluated for DMT1, aSyn, APP, DRD2, TH, and BDNF. Obese male D2J mice showed a significant upregulation of mRNA gene expression for DMT1, aSyn, APP, DRD2, and TH; and obese B6J female mice showed a significant upregulation of BDNF expression (Figure 4). Furthermore, there were significant diet-by-strain interaction effects for the expression of aSyn, DRD2, and TH, with the B6J strain showing a slight downregulation and the D2J strain showing a substantial upregulation in gene expression due to DIO. As mentioned above, this induction of mRNA in the D2J strain with DIO mainly affected male mice.

DMT1 acts as a transporter for Fe, Mn, Cu, and to a lesser extent Zn [40,41]. Considering the significant Fe and Mn alterations found in male B6J mice with DIO, we predicted an impact on DMT1 gene expression in B6J males as well. Unexpectedly, only D2J males were highly impacted, with a significant upregulation in DMT1 in the olfactory bulbs due to DIO. A previous study in our lab that examined the effects of DIO in male B6J mice showed that DIO did not induce DMT1 gene expression changes in other brain regions in male B6J mice [10]. However, a more recent DIO study from our lab comparing male and female B6J and D2J mice found a significant upregulation of DMT1 in the hippocampuses of male B6J mice, but found no changes in the gene expression of DMT1 in the striata [12]. These differences show that gene expression alterations due to DIO are brain region specific and are influenced by sex and strain. The effect of DIO on DMT1 expression in the context of Fe metabolism has been studied systemically [42,43,44], yet there is limited research focused on the brain. For example, in the duodenum, mRNA was upregulated more in DBA/2 mice fed an Fe-deficient diet compared with C57Bl/6 mice, while an Fe-supplemented diet resulted in a downregulation of DMT1 in DBA/2 mice, but had no effect on C57Bl/6 mice [45]. The effects of DIO on DMT1 gene expression reported in the literature have focused primarily on male rodents and the system, leaving a knowledge gap on DIO impact in the brain and the effect of sex on potential gene expression alterations. More investigations are needed to learn about the gene expression of DMT1 in the brain under DIO conditions, as the dysregulation of DMT1 and trace element homeostasis is associated with various neurodegenerative diseases [46,47].

We were interested in evaluating the impact of DIO on aSyn gene expression in the olfactory bulb for several reasons. A previous study from our lab showed an upregulation of aSyn, along with an increase in Fe, in the midbrain of male B6J mice due to DIO [10]. Accumulation of aSyn and Fe in the midbrain is also a hallmark of PD [48]. Furthermore, aSyn has been shown to accumulate in the olfactory bulb of PD patients [18,19]. There are several known biochemical interactions between aSyn and Fe in the brain [49,50]. Fe can regulate aSyn levels post-transcriptionally through the binding of iron regulatory protein on the aSyn iron response element, and post-translationally by interfering with the normal ubiquitination process of aSyn protein [49]. It has been proposed that aSyn can also regulate Fe levels through its ferroreductase activity, a process that depends on Cu as a cofactor. Overexpression of aSyn in both cell and animal models increases the intracellular reduction of ferric ion to ferrous ion, thereby increasing the risk for reactive oxygen species generation through the Fenton reaction. In the current study, we found a 1.6-fold upregulation of aSyn in male D2J mice and a significant diet-by-strain interaction. The expression of aSyn in the B6J strain was not impacted significantly by DIO. Unexpectedly, the increase in Fe that we found in B6J males in the olfactory bulb was not associated with an induction of aSyn mRNA in B6J males. Although Fe overload is known to promote aSyn aggregation [49], and aSyn upregulation has been associated with synucleinopathies [51], other studies have shown that aSyn mRNA expression is unchanged or reduced in PD brains, with Fe inducing aSyn protein synthesis at the translational level rather than the transcriptional level [52,53]. This may explain why we did not see an upregulation of aSyn mRNA in the olfactory bulbs of B6J mice fed an HFD, even though Fe was dramatically elevated in this brain region. In future studies, we will examine protein expression in addition to mRNA expression to determine whether elevated Fe in the olfactory bulb is associated with aSyn protein upregulation. This test was not performed in the current study due to lack of tissue availability. Regardless, this study reveals important strain and sex differences in response to an HFD for the expression of aSyn in the olfactory bulb, and should be further investigated for potential connections between DIO and neurodegenerative disease. 

APP is highly expressed in the olfactory bulb in rodents [54]. Our diet study included an analysis of APP gene expression since beta-amyloid aggregation in the olfactory bulb has been implicated in the progression of neurodegenerative disorders based on postmortem studies [16], and there is limited information on the effect of obesity or diet on APP expression in the olfactory bulb. Furthermore, the upregulation of APP is associated with AD [55]. In a study using Tg2576 AD mice, APP gene overexpression in the olfactory bulb impaired the function of protein kinase A between 6 and 18 months, leading the authors to speculate that this dysregulation of biochemical activities in the olfactory bulb supports the early progression of AD [56]. In the current study, APP mRNA was upregulated in male D2J mice fed an HFD by 2.7-fold, but was not significantly impacted in the B6J strain (Figure 4). Previous studies have also shown B6 and D2 strain differences in APP expression. For example, one study found that under conditions of stress, APP mRNA in the hypothalamus was upregulated in D2J mice but not in B6J mice [57]. Another study found that APP protein levels were upregulated in D2J glaucomatous retinas but not in B6 controls [58]. Our study provides novel information regarding the influence of genetics and the impact of DIO on APP gene expression in the olfactory bulb, which may be connected to neurodegenerative disease.

TH is a rate-limiting enzyme that requires Fe as a cofactor for the synthesis of catecholamines such as dopamine, epinephrine, and norepinephrine [59]. While several reports provide information about DIO-induced dysregulation of TH mRNA expression in the midbrain or TH protein expression in the striatum and midbrain, there is very little information regarding TH mRNA expression in the olfactory bulb in the context of DIO. In the present study, we found a significant 3.8-fold upregulation of TH mRNA in the olfactory bulbs of male D2J mice (Figure 4). This is consistent with previous rodent studies, which have reported an upregulation in TH mRNA expression due to DIO in other brain regions. For example, in a study using male C57BL/6 mice fed an HFD for 20 weeks, there was an upregulation of TH mRNA in the midbrains of mice fed an HFD, and a positive correlation between final body weight and TH gene expression in this brain region [60]. A study using female C57BL6/129SVJ mice fed an HFD for 12 weeks discovered an induction of TH mRNA expression in the hypothalamus using microarray analysis and real-time polymerase chain reaction techniques [61]. The induction of TH mRNA that we found in the olfactory bulbs of male D2J mice fed an HFD for 16 weeks indicates that the impact of DIO in this brain region is influenced by sex and strain. While we do not fully understand the neurophysiological mechanisms behind these sex and strain influences in TH gene expression, our preliminary findings provide a foundation for future work involving the impact of DIO on gene expression dysregulation in the brain.

DRD2 is expressed both pre- and postsynaptically in various brain regions, such as the striatum, midbrain, cortex, and olfactory bulb [62,63]. In the olfactory bulb, we found a significant 3.5-fold upregulation in DRD2 mRNA in male D2J mice and an estimated 2-fold downregulation in male B6J mice that was approaching significance (Figure 4). DIO did not impact the gene expression in females of either strain. To the best of our knowledge, there are no prior published results regarding the impact of DIO on DRD2 expression in the olfactory bulb. However, strain differences between B6J and D2J mice for the mRNA expression of DRD2 in the forebrain have been reported [64]. In a study using male and female offspring from female B6J mice bred with D2J males fed an HFD for 12 weeks, both males and females showed a downregulation of DRD2 mRNA in the nucleus accumbens core [65]. Another study using male B6J mice fed an HFD for 20 weeks found an induction of DRD2 mRNA in the nucleus accumbens cores of mice fed an HFD [60]. The opposite trends in mRNA dysregulation in the olfactory bulb for the B6J and D2J strains also highlight the important influence of genetics on the brain’s response to a state of DIO. 

The mRNA expression of BDNF has been identified in brain regions such as the hippocampus, cortex, and olfactory bulb [66], and dysregulated levels of BDNF have been associated with obesity [67]. We found a significant diet-by-sex interaction for the expression of BDNF in the olfactory bulb, which showed a greater impact on females compared with males. Alterations due to DIO were greatest for B6J females, with a 2.1-fold increase in BDNF mRNA expression for the obese group (Figure 4). Previous studies in rodent models have also shown an upregulation of BDNF mRNA or protein due to DIO; however, these results were in males and in different brain regions. For example, a study using male C57BL/6 mice fed an HFD for 8 weeks found that BDNF mRNA and protein were upregulated in whole-brain tissue and in HT-4 hippocampal neurons [67]. In a study using male Long–Evans rats fed an HFD for 72 h, the mRNA expression of BDNF was upregulated in the hippocampus [68]. In contrast, a study using female Fischer 344 rats fed a diet high in fat and sugar found that BDNF protein and mRNA expression in the hippocampus was reduced after 6 months of diet treatment [69]. To the best of our knowledge, our study is the first study to reveal DIO-induced alterations in BDNF mRNA expression in the murine olfactory bulb. As BDNF has an important role in neural plasticity and neuronal protection in several brain regions, including the olfactory bulb, this could have important health implications regarding the development of behavior disorders and neurodegenerative disease.

## 5. Conclusions

In sum, we found that DIO led to alterations in trace element homeostasis in the olfactory bulb predominantly in male B6J mice, and gene expression dysregulation in the olfactory bulb mainly in male D2J mice. Fe and Mn were significantly elevated only in B6J males fed an HFD. There was a significant three-way interaction between diet, sex, and strain for Zn, which showed an increase in Zn for B6J males and D2J females, but a decrease in Zn for B6J females and D2J males fed an HFD. There was a significant mRNA upregulation of DMT1, aSyn, APP, DRD2, and TH only in D2J males fed an HFD and a significant upregulation of BDNF in B6J females. Our hypothesis that DIO would cause alterations in Fe, Zn, and aSyn gene expression in the olfactory bulb was correct, although the results depended on sex and strain. The strengths of this study include the novel information regarding the impact of DIO on the olfactory bulb and the use of both male and female mice of two strains to reveal influences of sex and genetics. A limitation of this study was the lack of protein expression data due to limited tissue availability. In future studies, we will examine both mRNA and protein expression together to understand the downstream effects of gene expression alterations. Additionally, we will evaluate the impact of elevated olfactory bulb Fe and Mn in male B6J mice on murine behaviors. Overall, our results show that DIO has the potential to disrupt trace element homeostasis and mRNA gene expression in the olfactory bulb, with effects that depend on sex and genetics. These results have important implications for health outcomes related to obesity with possible connections to neurodegenerative disease.

## Figures and Tables

**Table 1:** Study design based on strain, sex, and diet.

	B6J		D2J	
	Male	Female	Male	Female
LFD	9	9	9	9
HFD	9	9	9	9

B6J = C57BL/6J, D2J = DBA/2J, LFD = low-fat diet, HFD = high-fat diet.
